# KRT13 and UPK1B for differential diagnosis between metastatic lung carcinoma from oral squamous cell carcinoma and lung squamous cell carcinoma

**DOI:** 10.1038/s41598-023-49545-9

**Published:** 2023-12-18

**Authors:** Hiroyuki Goda, Koh-ichi Nakashiro, Yoshifumi Sano, Tomoko Adachi, Norihiko Tokuzen, Nobuyuki Kuribayashi, Satoshi Hino, Daisuke Uchida

**Affiliations:** 1 Department of Oral and Maxillofacial Surgery, Ehime University Graduate School of Medicine, Toon, Japan; 2 Department of Cardiovascular and Thoracic Surgery, Ehime University Graduate School of Medicine, Toon, Japan

**Keywords:** Oral cancer, Cancer

## Abstract

Oral squamous cell carcinomas unusually show distant metastasis to the lung after primary treatment, which can be difficult to differentiate from primary squamous cell carcinoma of the lung. While the location and number of tumor nodules is helpful in diagnosing cases, differential diagnosis may be difficult even with histopathological examination. Therefore, we attempted to identify molecules that can facilitate accurate differential diagnosis. First, we performed a comprehensive gene expression analysis using microarray data for OSCC-LM and LSCC, and searched for genes showing significantly different expression levels. We then identified *KRT13*, *UPK1B*, and nuclear receptor subfamily 0, group B, member 1 (*NR0B1*) as genes that were significantly upregulated in LSCC and quantified the expression levels of these genes by real-time quantitative RT-PCR. The expression of *KRT13* and *UPK1B* proteins were then examined by immunohistochemical staining. While OSCC-LM showed no *KRT13* and *UPK1B* expression, some tumor cells of LSCC showed *KRT13* and *UPK1B* expression in 10 of 12 cases (83.3%). All LSCC cases were positive for at least one of these markers. Thus, *KRT13* and *UPK1B* might contribute in differentiating OSCC-LM from LSCC.

## Introduction

Globally, an estimated 354,864 new cases and 177,384 deaths occurred from oral cavity cancer in 2018^1^. The prognosis for patients with oral squamous cell carcinoma (OSCC) is determined by multiple factors, like primary tumor size, the presence of lymph nodes, distant metastases, and histopathological grade^2,3^. Control of distant metastasis in OSCC is an important determinant of an improved prognosis. Distant metastasis is observed only in about 4% of OSCC, of which approximately 90% are lung metastases^4^. Moreover, recent advancements in high-precision CT imaging have increased the frequency of identification of lung mass shadows and early detection of lung and metastatic lung cancer^5^. In patients with a history of OSCC, the differentiation between lung tumors metastasized from oral cancer and those originated from primary lung cancer is important. However, in most cases, these tumors are very difficult to differentiate clinically and pathologically. The morphologic features of lung metastasis of OSCC (OSCC-LM) and lung squamous cell carcinoma (LSCC) appear to be similar. The management and prognosis for OSCC-LM and LSCC are completely different, so distinguishing the two scenarios is important. In patients with head and neck SCC and solitary SCC in the lung, microsatellite analysis has been reported to be a genetic approach for identifying clonal relationships, but has yet to be applied clinically^6^. Furthermore, while next-generation sequencing technologies have facilitated the discovery of novel drivers in diverse types of SCCs, discerning clear distinctions among OSCCs, OSCC-LMs, and LSCCs remains challenging^7^. To date, no efficient marker to distinguish these two conditions has been proposed. Thus, in this study, we tried to investigate the identification of a novel effective marker being essential to facilitate a definite diagnosis.

## Materials and methods

### Samples from patients

OSCC-LM and LSCC tissues were obtained from patients at the Ehime University Hospital between June 2004 and June 2014. The study was approved by the Ehime University Hospital Ethics Committee (no.1106009), which confirms that all research was conducted in accordance with relevant guidelines/regulations. Informed consent was obtained from all participants and the study was conducted in accordance with the Declaration of Helsinki. The pathological and imaging diagnosis was confirmed by two senior specialists. The radiologist diagnosed the patient with LSCC and no other neoplastic lesions following a systemic search and with imaging findings characteristic of primary lung cancer, including spinous process and pleural intrusion. The radiologist diagnosed OSCC-LM based on a history of OSCC, and when the imaging findings generally showed a round or oval nodular shadow with relatively clear margins of the tumor shadow.

### Total RNA extraction

In the process of tumor sampling, we excised a segment measuring around 5 mm from the solid core of the tumor. For microarray and quantitative RT-PCR assays, total RNA was extracted from fresh sample tissues homogenized in 0.5 mL of ISOGEN (NipponGene, Tokyo, Japan), following the manufacturer’s instructions using TissueLyser (Qiagen, Valencia, CA). RNA integrity was confirmed using the Agilent 2100 Bioanalyzer (Agilent Technologies, Santa Clara, CA, USA). We verified that all RNA utilized possessed an RNA Integrity Number (RIN) of at least 2.0 and a DV200 value exceeding 50%.

### Microarray analysis

The Applied Biosystems Chemiluminescent RTIVT Labeling Kit (Life Technologies, Carlsbad, CA) was used to convert total RNA to digoxigenin (DIG)-labeled cRNA. Double-stranded cDNA was generated using 1 μg of total RNA, transcribed using DIG-labeled nucleotides (Roche Diagnostics, Basel, Switzerland), fragmented, and hybridized for Human Genome Survey Arrays (Life Technologies) following the manufacturer’s instructions. After washing each array, the signal was developed using a chemiluminescent detection kit (Life Technologies). Processed arrays were scanned on a 1700 chemiluminescent microarray analyzer (Life Technologies), and the results were analyzed using GeneSpring GX 13.0 (Agilent Technologies).

### Quantitative RT-PCR

To analyze target mRNA expression, cDNA was synthesized using a miScript II RT Kit (Qiagen), and quantitative RT-PCR (qRT-PCR) was performed using a miScript SYBR Green PCR Kit (Qiagen). The PCR amplification was conducted in a 10 µL final reaction volume containing 5 µL of 2 × QuantiTect SYBR Green Master Mix, 1 µL of 10 × miScript Universal Primer, 1 µL of 10 × miScript Primer Assay, 2 µL of RNase-free water, and 1 µL of cDNA. The thermal-cycling conditions were as follows: PCR initial activation step at 95 °C for 15 min, followed by 40 cycles of 94 °C for 15 s and 55 °C for 30 s. To analyze mRNA expression, the One Step SYBR PrimeScript RT-PCR Kit II (Takara) was utilized. PCR amplification was performed in a 10 μL final reaction mixture containing 5 μL of 2 × One Step SYBR RT-PCR Buffer 4, 0.4 μL of PrimeScript One Step Enzyme Mix 2, 0.4 μL of forward primer (10 μmol/L), 0.4 μL of reverse primer (10 μmol/L), and 100 ng of total RNA. Reverse transcription was performed at 42 °C for 5 min, followed by 95 °C for 10 s. The PCR amplification step comprised 40 cycles at 95 °C for 5 min and 60 °C for 10 s. The relative expression was calculated using ΔCt (cycle threshold) method and normalized to the control using the equation 2 − ΔΔCt. RNU6B and hydroxymethylbilane synthase (*HMBS*) were used as internal controls. SYBR Green 1 was detected using ViiA 7 (Thermo Fisher Scientific). The primer sequences used in this study were as follows: uroplakin 1B (*UPK1B*) forward, 5′- TGT TCG TTG CTT CCA GGG CCT GC -3′ and reverse, 5′- AGT AGA ACA TGG TAC CCA GGA GAA CC -3′, cytokeratin 13 (*KRT13*) forward, 5′- TTG AAA ACA ACC GGG TCA TC -3′ and reverse, 5′- GCT CTT CAT TCA GGC TCT CG -3′, nuclear receptor subfamily 0, group B, member 1 (*NR0B1*) forward, 5′- CCT CTC ACA GGC AGA ATG AAA T -3′ and reverse, 5′- TAT ACC AGC TGA TAC AGA ATC ATT -3′and *HMBS* forward, 5′-CAT GCA GGC TAC CAT CCA TGT C-3′ and reverse, 5′-GTT ACG AGC AGT GAT GCC TAC CAA-3′.

### Immunohistochemistry

The surgically resected specimens were fixed in phosphate-buffered saline (PBS) with 10% formalin and embedded in paraffin, and a series of tissue sections (thickness, 4 μm) were prepared from each sample. Immunohistochemical staining was performed by the avidin–biotin-peroxidase complex method. Briefly, the sections were deparaffinized, pretreated with 10 mM citrate buffer (pH 6.0) in an autoclave at 121˚C for 5 min, and incubated with 0.3% H_2_O_2_ in distilled water for 15 min to block endogenous peroxidase activity. After blocking with 3% horse serum in PBS, the sections were then incubated overnight at 4 °C with specific antibodies to human uroplakin 1b (polyclonal: diluted 1:500, Novus Biologicals, CO, USA) and human *KRT13* (monoclonal: diluted 1:500, Leica BIOSYSTEMS, Newcastle, UK). After washing, the sections were incubated with biotinylated anti-mouse/rabbit antibody (Vector Laboratories, Burlingame, CA) at room temperature for 30 min and washed in PBS, followed by labeling with streptavidin-peroxidase complex (Vector Laboratories). The peroxidase reaction was developed with 393-diaminobenzidine as a chromogen. The sections were counterstained with hematoxylin, dehydrated with ethanol, treated with xylene, and enclosed in synthetic resin. Quantitative studies of the immunohistochemically stained sections were performed by pathologists in a blinded fashion by evaluating three randomly chosen fields in each sample. Negative controls were set without the primary antibody incubation. Semiquantitative studies of the immunohistochemically stained sections were performed in a blind fashion by evaluating three randomly chosen fields in each sample. These samples were scored on the basis of immunoreactivity scoring system (IRS)^8^. The IRS was calculated by the product of the percentage of positive cells (4, > 80%; 3, 51–80%; 2, 10–50%; 1, < 10%; 0, 0%) and the intensity of the staining (3, strong; 2, moderate; 1, mild; and 0, no staining), which resulted in IRS scores between 0 (no staining) and 7 (maximum staining). The cancer cells stained with anti-*KRT13* and anti-*UPK1B* antibodies in cytoplasm were counted. In this study, positive cases were defined as the total IRS scores of *KRT13* and *UPK1B* at 3.5 or higher.

### Statistical analysis

RT-PCR experiments were performed in triplicate and repeated thrice. Student’s *t* test was used to determine the significance of differences between the groups, with values of P < 0.05 considered statistically significant. Statistical analyses were conducted using the GraphPad Prism software, version 5.04 (GraphPad Software).

### Institutional review board statement

The Institutional Review Board at Ehime University Hospital approved this study (no.1106009).

### Informed consent

Appropriate written informed consent was obtained from each patient.

### Limitations

The number of samples in the present study was small because of the rare entity of OSCC-LM. Moreover, the cases capable to harvest the materials are also rare, because lung metastasis in OSCC means terminal stage. We consider that the results will be of value for clinicians working on these tumors as well as other similar tumors that are difficult to distinguish. Expanding the sample size and conducting a multicenter study in the future could be effective in balancing out the biases and increasing the credibility of our study.

## Results

### Identification of overexpressed genes in LSCC by microarray analysis

**Figure 1:**
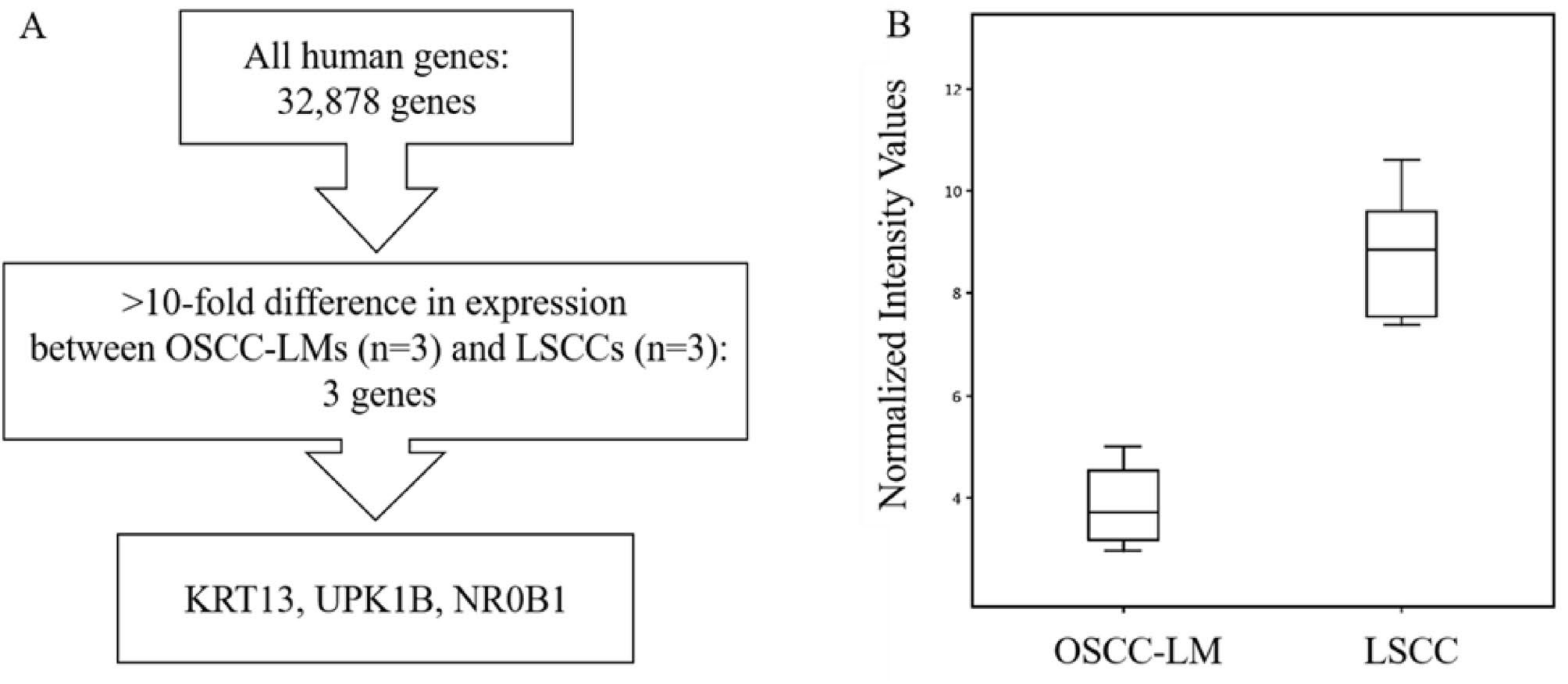
Identification of candidate molecular markers for differential diagnosis between OSCC-LMs and LSCCs by microarray analysis. (**A**) Experimental plan for microarray analysis. (**B**) Box plot of *KRT13*, *UPK1B*, and *NR0B1* mRNA expression levels in OSCC-LM and LSCC.

Using microarray analysis, we compared the expression profiles in three patients with LSCC and three patients with OSCC-LM. We found three genes that were overexpressed more than tenfold in all LSCCs in comparison with OSCC-LMs: *KRT13*, *UPK1B*, and *NR0B1* (Fig. 1 and Table 1).

**Table 1:** Identification of candidate molecular markers for differential diagnosis between OSCC-LMs and LSCCs by microarray analysis.

Gene symbol	Gene title	Fold change (OSCC-LM/LSCC)	p-value
*KRT13*	Keratin13	− 75.7	0.000243
*UPK1B*	Uroplakin 1B	− 39.8	0.000248
*NR0B1*	Nuclear receptor subfamily 0, group B, member 1	− 17.6	0.0000788

Genes exhibiting significantly higher expression in LSCC relative to OSCC-LM tissue were identified. (p-value: Student’s *t* test).

### Overexpression of KRT13 and UPK1B in LSCC by quantitative RT-PCR (qRT-PCR)

**Figure 2:**
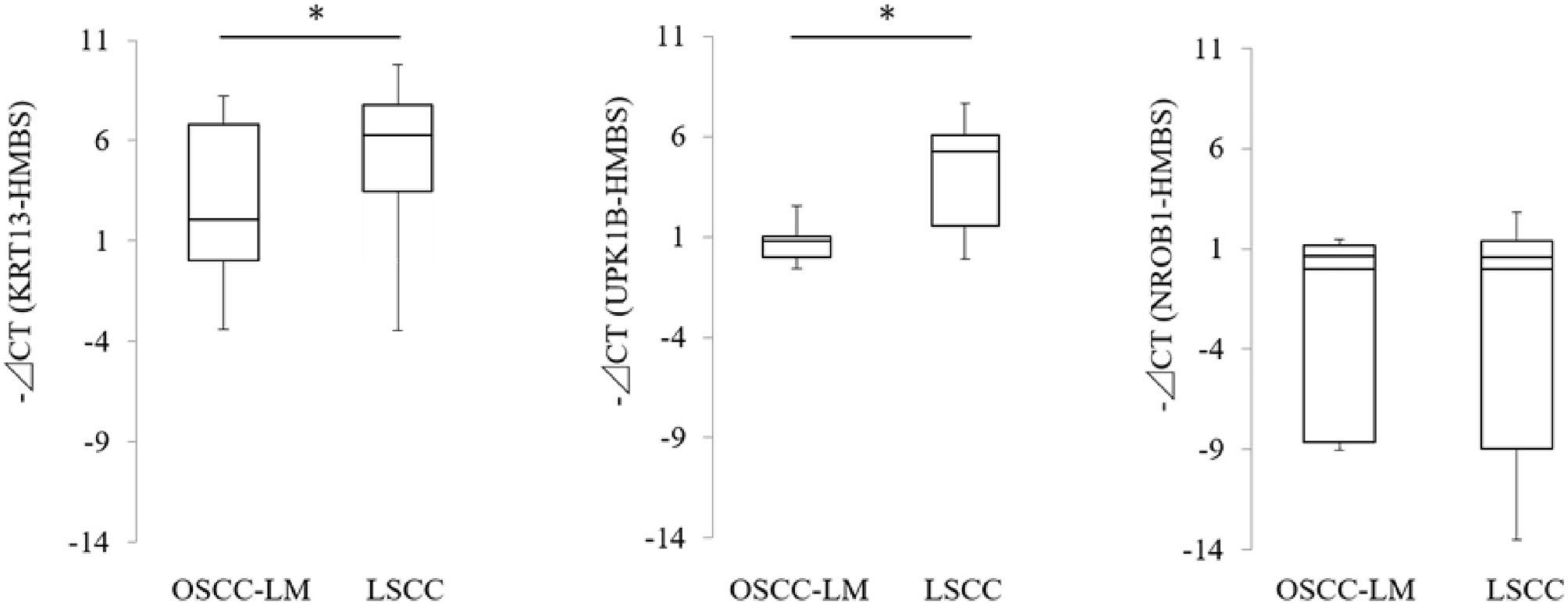
Quantification of candidate molecular markers in OSCC-LMs and LSCCs by qRT-PCR. Three genes, *KRT13*, *UPK1B*, and *NR0B1*, were evaluated by qRT-PCR using seven cases of OSCC-LM and eight cases of LSCC. *CT* cycle threshold; *p < 0.05.

We were able to design RT-PCR primers for *KRT13*, *UPK1B*, and *NR0B1*. The expressions of these three genes were examined by qRT-PCR in seven cases of OSCC-LM and eight cases of LSCC. Among the three genes, *KRT13* and *UPK1B* were significantly overexpressed in LSCCs (Fig. 2).

### Immunohistochemical staining profile of the top genes

**Figure 3:**
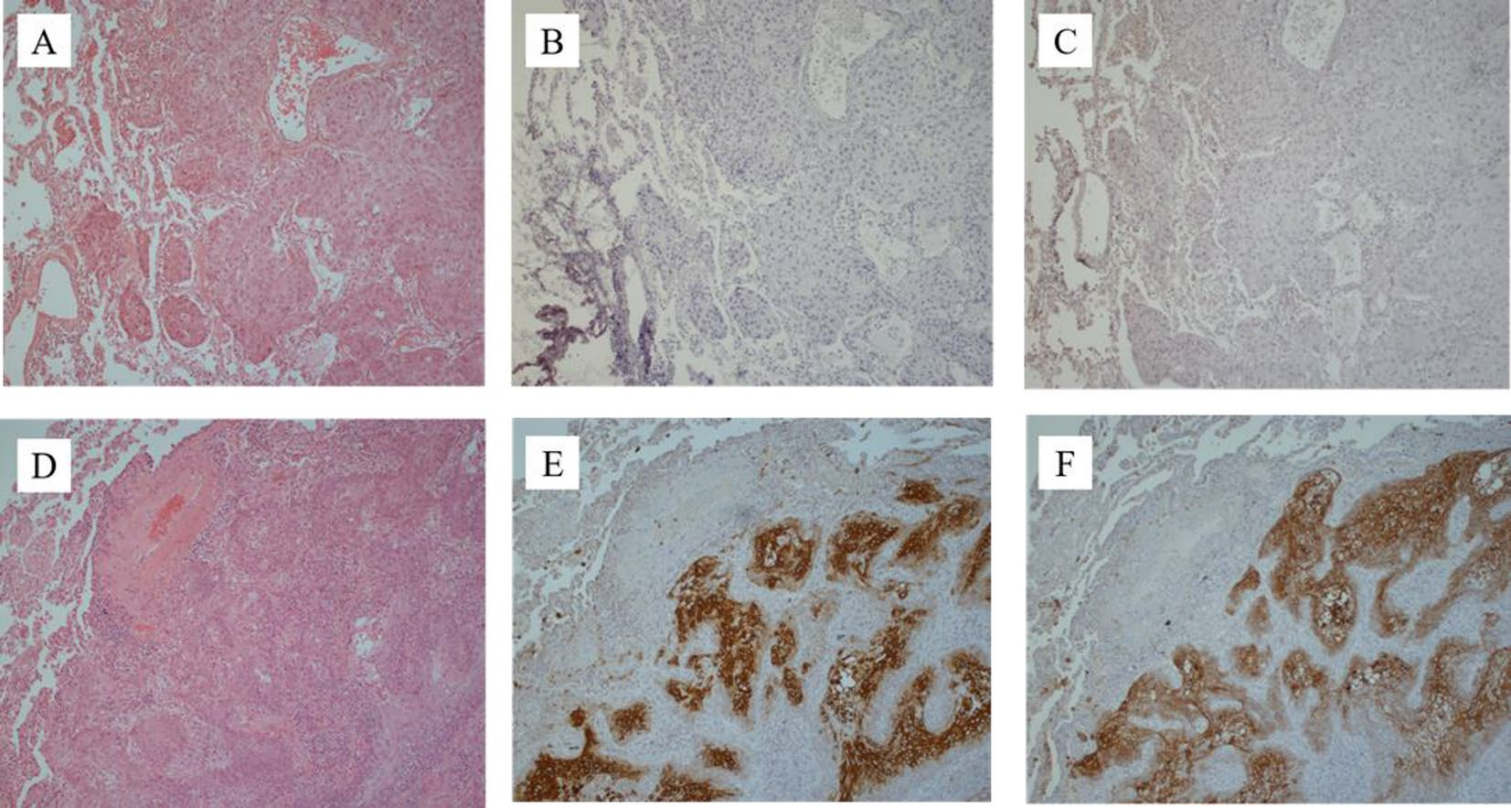
Representative immunohistochemical profiles of OSCC-LM ((**A**–**C**); Case No.5) and LSCC ((**D**–**F**); Case No.5). Photomicrograph showing hematoxylin–eosin staining (**A**,**D**). LSCC is positive for *KRT13* ((**B**), IRS 0; (**E**), IRS 6). LSCC is positive for *UPK1B* ((**C**), IRS 0; (**F**), IRS 6). Original magnification, × 10.

To confirm the significance of the upregulated genes, we performed immunohistochemical (IHC) staining for verification of protein expression and to determine the top two upregulated genes (*KRT13* and *UPK1B*) in LSCCs. To investigate the consistency of *KRT13* and *UPK1B* expression between OSCC-LMs and LSCCs, we analyzed 5 cases of OSCC-LMs and 12 cases of LSCC. *KRT13* and *UPK1B* staining in OSCC-LM tissue were both 0%, and the corresponding values in LSCC tissue were both 83.3%, when IRS ≥ 3.5 was evaluated as positive. Notably, all LSCC cases showed positive results for at least one of the markers (Fig. 3 and Table 2).

**Table 2:** The expression of *KRT13* and *UPK1B* in OSCC-LMs and LSCCs.

Case no	OSCC-LMs					Positive rate	LSCCs												Positive rate
	1	2	3	4	5		1	2	3	4	5	6	7	8	9	10	11	12	
KRT13	–	–	–	–	–	0% (0/5)	–	+	+	+	+	–	+	+	+	+	+	+	83.3% (10/12)
% of P.C	0	0	0	0	0		0	2.7	3	2.7	3	0	3	3	3.3	3.3	4	3.7	
Intensity	0	0	0	0	0		0	3	3	1	3	0	2.3	1.7	2.3	2.3	2	1.7	
IRS	0	0	0	0	0		0	5.7	6	3.7	6	0	5.3	4.7	5.7	5.7	6	5.3	
UPK1B	–	–	–	–	–	0% (0/5)	+	–	–	+	+	+	+	+	+	+	+	+	83.3% (10/12)
% of P.C	0	0	0	0	0		4	2	2.3	3.3	3	4	3	2.7	3	3.3	4	4	
Intensity	0	0	0	0	0		2	1	1	2	3	3	2	1	1.3	3	3	1	
IRS	0	0	0	0	0		6	3	3.3	5.3	6	7	5	3.7	4.3	6.3	7	5	

## Discussion

Head and neck cancers, including oral cancers often show double or secondary tumors in the lung at the rate of 5%^9,10^. In such cases, both cancers are often squamous cell carcinomas, and the difficulty in distinguishing between metastasis and primary lung cancer is problematic because both entities require different treatment strategies and show differences in prognosis. In previous reports, patients with T1 or T2 head and neck cancer without local recurrence were considered to have primary lung cancer, while those with neck lymph node metastasis were considered to have metastatic lung cancer^9^. Lefor et al. reviewed 55 cases of head and neck cancer combined with lung lesions and found that in cases where histological discrimination was difficult, a single lung lesion and negative cervical lymph node enlargement were the criteria for primary lung cancer. They also reported that 11 of the 40 cases occurred within 6 months, and the short duration does not necessarily mean that the lung lesion represents metastasis^11^. There is little scientific evidence for either of these conclusions, and no method has been established to strictly differentiate between the two forms of lung tumors. Since the commencement of human genome projects, molecular analysis has become much easier. Now we can monitor expression of most genes simultaneously and comprehensively using microarray technologies. We have previously reported the results of our analysis of oral squamous cell^12–14^. Methods to identify the primary tumor site using genetic information of tumor tissues by microarray analysis and next-generation sequencing have been reported recently^15–17^. Microarray analysis has been suggested to be useful for accurately identifying the primary tumor site in 80% of solid tumors with a confirmed primary tumor site by evaluation of gene expression^15,16^. The Tissue of Origin test has been approved for use in the United States. In a prospective study of the Tissue of Origin test using 157 formalin-fixed paraffin-embedded samples with known primary sites, the microarray method was as diagnostic as immunostaining, and the microarray method was particularly useful in the diagnosis of poorly differentiated or undifferentiated cancers^18^. However, diagnosis by these genetic analyses is complex and expensive.

In this study, we identified *KRT13* and *UPK1B* as candidate molecular markers for differentiating OSCC-LM from LSCC. The keratins are intermediate filament proteins responsible for the structural integrity of epithelial cells. *KRT13* is a member of the keratin family, which plays a role in cytoskeleton remodeling. *KRT13* has been reported to be less expressed in cancerous tissues than in normal tissues in the oral mucosa^19,20^. Actually, according to a microarray data from the GEO profiles database (NCBI GEO database, https://www.ncbi.nlm.nih.gov/geo/tools/profileGraph.cgi?ID=GDS1584:207935_s_at, 2022–12-26), 4.5-fold downregulation of *KRT13* in OSCC tissues were observed in compared with that in normal oral mucosa. On the other hands, Bloor and colleagues^21^ demonstrated that expression of *KRT13* in the well-differentiated primary OSCC was predominantly detected than that in moderately-differentiated OSCC. In contrast, expression of both mRNA and protein expression of *KRT13* was not detected in the poorly differentiated OSCC, which generally shows more aggressive and highly metastatic potentials. Thus, downregulation of *KRT13* is suggested to be used as a marker for malignant potential and differentiation of stratified squamous epithelium^21,22^. In the present study, *KRT13* gene expression was predominantly down-regulated in OSCC-LM in comparison with LSCC, and no *KRT13* protein expression was observed in OSCC-LM. These findings might support that more malignant cancer cells without *KRT13* expression in the primary sites predominantly metastasize to the lung, resulted in the loss of *KRT13* expression in the OSCC-LM.

Although there were no direct comparative reports of *KRT13* expression in comparison between OSCC-LM and LSCC, expression of *KRT13* in LSCC showed a very high signal value than that in normal lung and lung adenocarcinoma (LAdC) from the GEO profiles database (NCBI GEO database, https://www.ncbi.nlm.nih.gov/geo/tools/profileGraph.cgi?ID=GDS1312:207935_s_at, 2022–12-26, NCBI GEO database, https://www.ncbi.nlm.nih.gov/geo/tools/profileGraph.cgi?ID=GDS3627:207935_s_at, 2022–12-26). Moreover, Zhan and colleagues also reported that expression of *KRT13* in LSCC was greatly elevated that in LAdC at the fold change of 83.76 from the 490 each samples of LSCC and LAdC by analyzing TCGA database^23^. These data are indicating the specificity of *KRT13* expression in LSCC among non-small cell lung cancers. The utility of *KRT13* as a marker in this study may be due to the possibility that less differentiated and more malignant cells in oral squamous cell carcinoma are responsible for lung metastasis.

*UPK* families play an important role in maintaining uroepithelium function, and five of these proteins have been identified to date: *UPK1A*, *UPK1B*, *UPK2*, *UPK3A* and *UPK3B*. *UPK1A* and *UPK1B* are tetraspanin proteins and possess four transmembrane domains, whereas *UPK2* and *UPK3* have single transmembrane domains^24^. *UPK1B* has been reported to be closely associated with tumor development, progression, and chemotherapy resistance in bladder, gastric and colorectal cancers. However, the molecular mechanisms underlying the function of *UPK1B* in tumors have not been fully elucidated^25–29^. However, UPK1B is also poorly expressed in oral mucosa and OSCC, suggesting that it is unlikely to be an oncogenic molecule. Conversely, its expression is somewhat elevated in LSCC, which could indicate its oncogenic potential in this setting. (THE HUMAN PROTEIN ATLAS: https://www.proteinatlas.org/ENSG00000114638-UPK1B) It is also theorized that some LSCC may originate de novo from the bronchoalveolar epithelial system, or from squamous metaplasia or dysplasia^30^. Therefore, it is possible that the results may reflect the expression of UPK1B in the glandular epithelium. Large-scale tissue microarray analyses by Reiswich et al. showed that positive expression of *UPK1B* was detectable at the rate of 39% in LSCC, but was only 18% in OSCC arised in oral floor^31^. They also described that *UPK1B* positivity rates varied markedly between SCC of different organs, and recommended the limited utility in case of SCC metastases from unknown primary tumors. Their opinion might support our results for the use of metastatic marker on OSCC, although the interpretation of the result for *UPK1B* need to be cautious. Further studies for *UPK1B* expression on the primary sites in OSCC (e.g., tongue, gingiva etc.) might be clarified the mechanism of its regulation. A more comprehensive analysis of the molecular mechanisms is required to ascertain the efficacy of *UPK1B* as a differential marker.

In the present study, the positive rate of both markers in LSCC was relatively high at the rate of approximately 80%. Similar to our findings, upregulation of *KRT13* mRNA and *UPK1B* mRNA in LSCC were frequently detectable at the positive rate of 77.8% and 61.1%, respectively, in the GEO profiles database using microarray analysis (NCBI GEO database, https://www.ncbi.nlm.nih.gov/geo/tools/profileGraph.cgi?ID=GDS3627:207935_s_at, 2022–12-26, NCBI GEO database, https://www.ncbi.nlm.nih.gov/geo/tools/profileGraph.cgi?ID=GDS3627:210064_s_at, 2022–12-26). In contrast, large scale tissue microarray analysis in LSCC using IHC showed that expression of *KRT13* and *UPK1B* protein were detected only in 63% and 39%, respectively^32^. Although the reason on the different positive rate of these molecules is unclear, the difference of the antibody (monoclonal vs polyclonal) might lead the distinct results. The polyclonal antibody used in the present study might bind to multiple epitopes on the target protein, resulted in a higher signal.

In the present study, none of *KRT13* and *UPK1B* staining in OSCC-LM tissue were detectable, and all LSCC cases showed positive results for at least one of the markers. Although acquired data of OSCC-LM was limited because of the rare entity and difficulty of sample collection, our data suggesting that a combination of *KRT13* and *URK1B* may be helpful to differentiate OSCC-LMs from LSCCs.

## Conclusions

While further studies are needed, our data suggest that staining for *KRT13* or *UPK1B* might contribute to differentiate OSCC-LMs from LSCCs, which would help determine the appropriate treatment rapidly and cost-effectively and may contribute to patient prognosis.

## Data Availability

The data presented in this study are available on request from the corresponding author.
